# Adipose Tissue-Derived Extracellular Vesicles and the Tumor Microenvironment: Revisiting the Hallmarks of Cancer

**DOI:** 10.3390/cancers13133328

**Published:** 2021-07-02

**Authors:** João Alfredo Moraes, Carol Encarnação, Victor Aguiar Franco, Luiz Gabriel Xavier Botelho, Gabriella Pacheco Rodrigues, Isadora Ramos-Andrade, Christina Barja-Fidalgo, Mariana Renovato-Martins

**Affiliations:** 1 Redox Biology Laboratory, Programa de Pesquisa em Farmacologia e Inflamação, Instituto de Ciências Biomédicas, Universidade Federal do Rio de Janeiro, 21941-902 Rio de Janeiro, Brazil; joaomoraes@icb.ufrj.br; 2 Laboratory of Inflammation and Metabolism, Departamento de Biologia Celular e Molecular, Instituto de Biologia, Universidade Federal Fluminense, 24210-201 Niterói, Brazil; carolcosta@id.uff.br (C.E.); victorfranco@id.uff.br (V.A.F.); lugabrielbotelho@id.uff.br (L.G.X.B.); gabriellapacheco@id.uff.br (G.P.R.); 3 Laboratory of Cellular and Molecular Pharmacology, Departamento de Biologia Celular, IBRAG, Universidade do Estado do Rio de Janeiro, 20550-170 Rio de Janeiro, Brazil; andrade.isadora@posgraduacao.uerj.br (I.R.-A.); barja-fidalgo@uerj.br (C.B.-F.)

**Keywords:** extracellular vesicles, cancer, adipose tissue, tumor microenvironment, hallmarks of cancer

## Abstract

**Simple Summary:**

Increased body fat is associated with an increased risk of 13 different cancer types. Recent findings have demonstrated a close relationship between extracellular vesicles released by adipose tissues and the establishment and progression of several types of cancers and metastasis. However, detailed information about the establishment of such cooperation is still lacking. We provide evidence to support that extracellular vesicles secreted by adipose tissues may carry tumoral molecules that modulate the behavior and functions of cancer cells, as described in the seminal report “The Hallmarks of Cancer” by Hanahan and Weinberg, published in the early 2000s.

**Abstract:**

Extracellular vesicles (EVs) are crucial elements that sustain the communication between tumor cells and their microenvironment, and have emerged as a widespread mechanism of tumor formation and metastasis. In obesity, the adipose tissue becomes hypertrophic and hyperplastic, triggering increased production of pro-inflammatory adipokines, such as tumor necrosis factor α, interleukin 6, interleukin 1, and leptin. Furthermore, obese adipose tissue undergoes dysregulation in the cargo content of the released EVs, resulting in an increased content of pro-inflammatory proteins, fatty acids, and oncogenic microRNAs. These alterations drive obesity-associated inflammatory responses both locally and systemically. After being ignored for a long time, adipose tissues have recently received considerable attention as a major player in tumor microenvironment-linked obesity and cancer. The role of adipose tissue in the establishment and progression of cancer is reinforced by its high plasticity and inflammatory content. Such a relationship may be established by direct contact between adipocytes and cancer cells within the microenvironment or systemically, via EV-mediated cell-to-cell communication. Here, we highlight cues evidencing the influence of adipose tissue-derived EVs on the hallmarks of cancer, which are critical for tumor malignancy.

## 1. Introduction

Obesity is associated with an increased risk for several types of cancer. Apart from the typical risk factors such as genetic predisposition, tobacco use, unhealthy diet, and sedentary lifestyle [1], obesity is clearly linked to an overall increased risk for cancers and is related to a worse prognosis in patients with cancer [2]. It is estimated that 40% of cancer-related deaths are attributable to obesity [3]. This close association is further reinforced by epidemiologic reports showing that weight loss reduces cancer incidence [4]. Furthermore, increased body fat is correlated with an increased risk of 13 different types of cancer [5].

Chronic inflammation, a well-established characteristic of obesity, is a central component of tumor development and progression [6,7,8] and is characterized by metabolic dysregulation and/or reprogramming [9]. The role of adipose tissue in cancer is reinforced by the following attributes: (i) adipocytes are major components of the tumor microenvironment from tumors that are capable of metastasizing to the abdominal areas in breast, gastric, ovarian, and colon cancers; (ii) adipose tissues and the tumor microenvironment share the presence of inflammatory cells, especially macrophages [8]; (iii) adipose tissues are capable of recruiting immune cells [10]; (iv) secretion of pro-angiogenic molecules [11]; (v) adipose tissues provide excess substrate for adenosine triphosphate (ATP) production and lipid membrane generation [9,12,13]; (vi) adipose tissues secrete large amounts of extracellular vesicles (EVs), thereby mediating cell-to-cell communication [14,15].

In the past, studies focusing on the communication between adipocytes and tumor cells were limited to soluble mediators such as leptin or proinflammatory cytokines [16]. However, EVs have emerged as crucial elements underlying tumor formation, progression, and metastasis [17]. Evidence suggests that EVs sustain bidirectional communication between tumor cells and their microenvironment that is crucial for tumor occurrence and progression [18]. Circulating exosomes or microvesicles isolated from patients with cancer have been associated with metastasis or recurrence. Therefore, EVs are regarded as potential markers for cancer diagnosis and prognosis [19]. Circulating EV levels are significantly higher in obese patients [20]. There is a constant exchange of information between cancer cells and cells in the adipose tissue, mediating exchange of molecules such as non-coding RNAs, and changing the transcriptomic profile of both cells toward an oncogenic profile [21].

In the early 2000s, Hanahan and Weinberg in their seminal paper entitled “The Hallmarks of Cancer” [22] highlighted six biological capabilities that characterize tumor development, including sustained proliferative signaling, evasion of growth suppressors, resistance to cell death, acquisition of replicative immortality, induction of angiogenesis, and activation of invasion and metastasis. Genome instability and inflammation were described as processes that accelerate and promote these hallmarks.

In 2011, Hanahan and Weinberg revisited the hallmarks and proposed that tumors consist of more than just proliferative cancer cells; in fact, they are complex tissues composed of distinct cells types capable of interacting with each other in the so-called tumor microenvironment, which plays an active role in tumorigenesis, allowing the development of certain hallmark characteristics. Consequently, two other emerging hallmarks including dysregulation of cellular metabolism and evasion of immune destruction were added to this list [23].

It is well established that EVs derived from tumor cells can transform non-tumor cells or confer them with metastatic ability. EVs derived from MDA-MB-231 breast tumor cells induced epithelial-mesenchymal transition (EMT) in MCF-10 breast epithelial cells [24]. EVs derived from another triple-negative breast tumor cell (HCC1806) induced proliferation and drug resistance in MCF-10 cells through the alteration of genes and microRNAs (miRNAs) related to proliferation, invasion, and migration pathways [25]. Shen et al. showed that highly metastatic HO8910PM ovarian tumor cells can transfer EVs enriched with CD44 to poorly metastatic HO8910 cells, thereby increasing their malignancy [26]. Furthermore, Sakha et al. observed that exosomes derived from the highly metastatic human oral cancer cell line HOC313-LM can transfer microRNA (miR)-1246 to the poorly metastatic cancer cell line HOC313-P, leading to an increase in its mobility and invasion [27]. However, the focus of our review is to highlight the crosstalk between EVs derived from adipose tissues (AT-EVs) and tumor cells.

**Figure 1:**
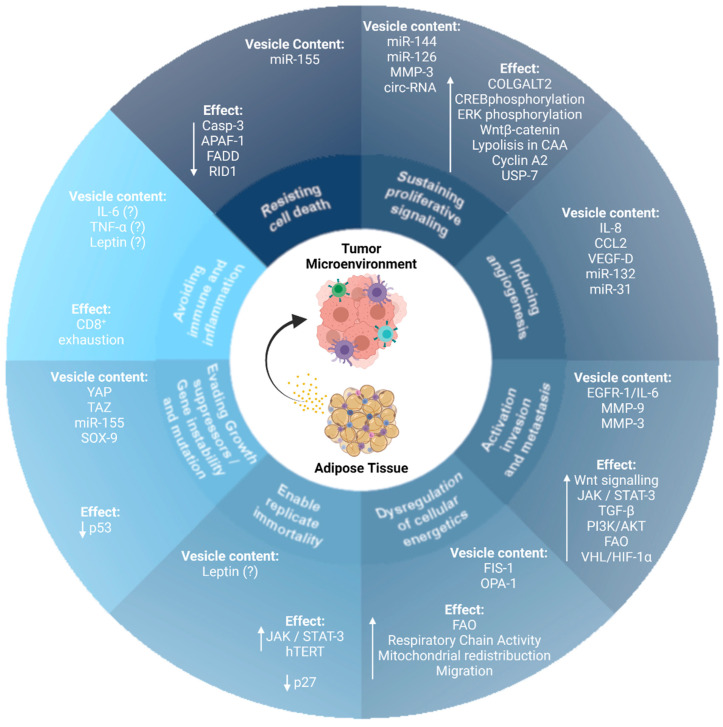
Extracellular vesicles (EVs) derived from adipose tissues modulate the acquisition and maintenance of cancer hallmark traits. EVs derived from adipose tissue modulate the acquisition and maintenance of the hallmark traits of cancer. MicroRNA (miR); circulating-RNA (circ-RNA); procollagen galactosyltransferase 2 (COLGALT2); cAMP response element-binding protein (CREB); CAA (cancer-associated adipocytes); ubiquitin-specific-processing protease 7 (USP-7); interleukin-8 (IL-8), C-C motif chemokine ligand 2 (CCL2), and vascular endothelial growth factor-D (VEGF-D); epidermal growth factor receptor 1 (EGFR1); interleukin 6 (IL-6); metalloproteinase 3 (MMP-3); metalloproteinase 9 (MMP-9); transforming growth factor β (TGF-β); janus kinase (JAK); signal transducer and activator of transcription 3 (STAT-3); hypoxia-inducible factor-1 α (HIF1-α); von-Hippel Lindau tumor suppressor (VHL); mitochondrial fission protein 1 (FIS-1); mitochondrial dynamin-like GTPase (OPA-1); fatty acid oxidation (FAO); human telomerase reverse transcriptase (hTERT); yes-associated protein 1 (YAP); taffazin (TAZ); caspase 3 (Casp-3); apoptotic peptidase activator factor 1 (APAF-1); Fas-associated death domain protein (FADD); GTPase-binding protein rid1 (RID1); SRY-box transcription factor 9 (SOX-9).

Importantly, AT-EVs modulate several cell biological capabilities that are characteristic hallmarks of cancer (Figure 1). In this review, we discuss how AT-EVs modulate the acquisition and maintenance of the hallmarks of cancer, providing critical biochemical and biomechanical cues that direct growth, migration, invasion, metabolic reprogramming, immune function, and metastasis of cancer cells. 

## 2. EVs

EVs are secreted by all cell types. The term EV was initially used to refer to a process used by cells to eliminate unnecessary components [28]. Nevertheless, research in the field has progressed rapidly leading to the discovery that EVs mediate cell-to-cell communication through the exchange of DNA, messenger RNAs (mRNAs), miRNAs, proteins, and lipids in a paracrine or systemic manner [29].

The term EV encompasses membrane vesicles of different sizes and origins that can be classified into three main categories: exosomes, microvesicles, and apoptotic bodies. The term exosome refers to membrane vesicles ranging between 30 and 100 nm in diameter that are generated by the inward budding of the endosomal membrane during multivesicular endosome maturation and are secreted onto the cell surface through the fusion of MVEs with the plasma membrane [30,31]. Since the 1990s, exosomes derived from B lymphocytes and dendritic cells have been reported to be associated with antitumor immune responses through immune regulation [32,33]. Microvesicles were first described as subcellular material that originated from platelets [34], and their functions were first shown to be related to blood coagulation. More recently, they have been shown to have important roles in cell-to-cell communication between various cell types and cancer cells. Microvesicles are generated by outbound budding and fission of the plasma membrane and range in size from 50 to 1000 nm. Apoptotic bodies derived from cells that undergo apoptosis are larger than 5 µm [35]. However, in this review, EVs are used to refer to only exosomes and microvesicles. EV cargo reflects the physiological or pathological microenvironment of its donor cell, allowing cell-to-cell communication through the exchange of its content, modulating the phenotype, and inducing novel properties in recipient cells through changes in intracellular signaling [36].

In the late 1970s, the secretion of EVs from spleen nodules and lymph nodes from a patient with Hodgkin’s disease drew research interest in the role of EVs in cancer [37].

### AT-EVs and Their Role in the Tumor Microenvironment

**Figure 2:**
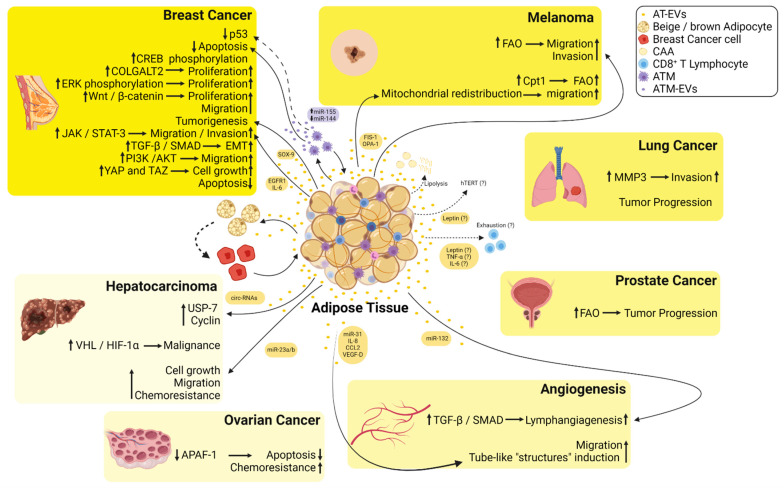
Summary of the crosstalk between the EVs derived from adipose tissues (AT-EVs) and several types of cancers. EVs released from adipose tissue macrophages are enriched in miR-155, whereas miR-144 is downregulated inducing inhibition of p53 and decreased apoptosis in breast cancer cells. AT-EVs increase phosphorylation of cAMP response element-binding (CREB) protein increasing the proliferation of breast cancer cells. AT-EVs increase the proliferation of breast cancer cells via induction of procollagen galactosyltransferase 2 (COLGALT2). AT-EVs increase extracellular signal-regulated kinase (ERK) and Wnt/β-catenin signaling pathways inducing an increase in the proliferation of breast cancer cells. AT-EVs enriched in SRY-box transcription factor 9 (SOX-9) induce tumorigenesis in breast cancer cells. AT-EVs from diabetic patients are enriched in epidermal growth factor receptor 1 (EGFR1)/interleukin 6 (IL-6) and induce an increase in migration and invasion of breast cancer cells via Janus kinase (JAK)/signal transducer and activator of transcription 3 (STAT-3) signaling. AT-EVs increase TGF-β/SMAD signaling pathway triggering epithelial-mesenchymal transition (EMT) in non-metastatic breast cancer cells. AT-EVs induce PI3K/AKT signaling in MDA-MB-231 cells, thus increasing their migratory capacity. AT-EVs induce yes-associated protein 1 (YAP) and taffazin (TAZ) inducing cell growth in breast cancer cells. AT-EVs decrease apoptosis in breast cancer cells. AT-EVs enriched in mitochondrial fission protein 1 (FIS-1) and mitochondrial dynamin-like GTPase (OPA-1) induce mitochondrial redistribution toward the edge of melanoma cells increasing their migratory capacity. AT-EVs and EVs derived from cancer-associated adipocytes (CAA) fuel free fatty acids to melanoma cells and increase fatty acid oxidation (FAO) increasing their migratory and invasive capacities. AT-EVs increase metalloproteinase 3 (MMP-3) in lung cancer cells increasing their invasiveness. AT-EVs increase tumor progression of lung cancer. AT-EVs increase FAO and induce tumor progression in prostate cancer. AT-EVs enriched in circulating RNAs (circ-RNAs) induce hypophosphorylation of ubiquitin-specific-processing protease 7 (USP-7) and an increase in cyclin in hepatocarcinoma cells. AT-EVs induce malignancy of hepatocarcinoma cells via hypoxia-inducible factor 1 α (HIF-1α) binding to the von-Hippel Lindau tumor suppressor (VHL). AT-EVs decrease apoptosis and increase chemoresistance in ovarian cancer cells via inhibition of the apoptotic peptidase activator factor 1 (APAF-1). AT-EVs enriched in miR-31, interleukin 8 (IL-8), C-C motif chemokine ligand 2 (CCL2), and vascular endothelial growth factor-D (VEGF-D) induce the formation of tube-like structures by endothelial cells. AT-EVs enriched in miR-132 induce lymphangiogenesis via transforming growth factor β (TGF-β)/Smad signaling. CAA undergo increased lipolysis and differentiate toward beige/brown adipocytes phenotype fueling tumor cells with EVs. Leptin released from AT is possibly within EVs and induces an increase in telomerase reverse transcriptase (hTERT) activity inducing resistance to apoptosis and senescence. Leptin, TNF-α, and IL-6 may be released from AT-EVs, inducing CD8^+^ T cells’ exhaustion.

Adipose tissue serves as a reservoir of proinflammatory adipokines in obesity that function not only in a paracrine manner but also have a systemic effect in enabling communication with distant sites. Several adipokines, including tumor necrosis factor α (TNFα), interleukin (IL) 6, IL-8, and chemokine (C-C motif) ligand (CCL) 2 have been shown to play a role in tumor progression [11,38]. Given that the increased levels of these pro-inflammatory cytokines are directly correlated with the volume of both visceral and subcutaneous adipose tissue depots in obese patients, this may explain how these adipose tissue depots fuel the tumor microenvironment. Adipose tissue reinforces tumor progression in obesity [8,11,39]; for example, once in the tumor microenvironment, adipocytes are delipidated, possibly fueling breast cancer cells and overexpressing matrix metalloproteinase (MMP) 11, supporting the invasion of breast cancer cells [40]. However, there is still a lack of information on the establishment of such cooperation. Although most studies have shown that the proximity of adipocytes and tumor cells induces adipocyte lipolysis by characterizing the role of adipocytes in later stages of cancer progression [41,42,43,44], it is currently accepted that adipocytes can trigger modifications in distant tumor cells by exchange of EVs through circulation [45] (Figure 2). Reinforcing this idea, Hartwig et al. demonstrated that adipokines are released either directly or via EVs, as the so-called exoadipokines, and constitute an essential part of the human adipose tissue secretome [46]. We previously demonstrated that adipose tissue from obese individuals secreted high amounts of EVs, which could modify the in vitro biological capabilities of breast cancer cells toward a more aggressive phenotype through intracellular signaling modifications sustained by cell-to-cell communication [15].

## 3. AT-EVs and the Hallmarks of Cancer 

### 3.1. Resisting Cell Death

An important hallmark of cancer cells is their ability to evade apoptosis, both in situations of nutrient deprivation and in the presence of chemotherapeutic drugs [23]. In this context, Wang et al. showed that human exosomes derived from mesenchymal stem cell (MSC)-differentiated adipocytes protected breast cancer cells from apoptosis induced by serum starvation and treatment with the chemotherapeutic drug, 5-fluorouracil. Furthermore, the authors showed that adipocyte-conditioned medium devoid of exosomes was unable to protect breast cancer cells from apoptosis [47]. In contrast, Reza et al. demonstrated that exosomes from adipose tissue-derived MSCs (ADMSCs) contain several miRNAs that induce the expression of the pro-apoptotic protein B-cell lymphoma 2 (Bcl-2) associated protein X (Bax), and caspase-9 in SKOV-3 and A2870 ovarian cancer cells, thereby impairing cell proliferation, wound repair, colony-forming capacity, and cell survival [48].

The interplay between adipose tissue and breast cancer is modulated by exosomes from adipose tissue macrophages [21]. In obese conditions, these exosomes are enriched in miR-155, which is involved in insulin resistance [49]. MiR-155 plays an oncogenic/anti-apoptotic role in breast cancer cells through caspase-3, Fas-associated death domain (FADD), receptor-interacting protein 1 (RIP-1), apoptotic peptidase activation factor-1 (APAF-1), and Bcl-2 [50]. Additionally, in MCF-7 breast cancer cells, miR-155 targets the tumor protein P53 inducible nuclear protein 1 (TP531NP1), conferring resistance to cell death [51]. The miRNAs found in exosomes can also act as tumor suppressors by maintaining homeostasis through cell-to-cell communication. For example, miR-148b, released into exosomes by adipose tissue, acts as a tumor suppressor in breast cancer cells. However, in obese conditions, these miRNAs are downregulated, and their pro-apoptotic activity is suppressed, reinforcing the role of obesity-associated adipose tissue in malignant transformation [21]. Together, these data indicate that exosomes secreted by adipose tissues actively participate in cell homeostasis in healthy conditions; however, in obese conditions, they may undergo changes in their cargo content that may protect tumor cells from apoptosis and increase cancer progression.

### 3.2. Sustaining Proliferative Signaling

One of the main features of cancer cells is their ability to sustain chronic proliferation, which is caused by the dysregulation of the production and release of growth-promoting signals. In addition, evasion of growth suppressors and resistance to cell death further contribute to their survival and tumor progression [23].

In the tumor microenvironment, interactions between the stroma and tumor cells are fundamental for tumor progression [52]. Exosomes play a key role in this communication, linking the diseased and healthy cells [53]. As mentioned above, exosomal crosstalk between adipose tissue and cancer cells may be a significant aspect of tumor progression by reprogramming the surrounding cells [54]. 

One of the mechanisms by which exosomes from ADMSCs induce cancer cell proliferation is through the modulation of Wnt/β-catenin signaling. Metastatic osteosarcoma is enriched in MSC-adipocytes and in vitro studies suggest that exosomes from ADMSCs increase proliferation and growth of osteosarcoma cells via the procollagen galactosyltransferase 2 (COLGALT2) pathway [39]. 

Jeurissen et al. demonstrated that treatment of breast cancer cells (ZR75.1) with cancer-associated AT-EVs increased the phosphorylation of cAMP response element-binding protein (CREB) [55]. Other studies have shown that the co-cultivation of breast cancer cells with adipocytes increases the expression of fatty acid transporters, thereby sustaining increased proliferation [56]. Ramos-Andrade et al. showed that increased proliferation of MCF-7 cells in vitro after treatment with EVs secreted by the obese adipose tissue involves the activation of the extracellular signal-regulated kinase (ERK)/mitogen-activated protein kinase (MAPK) signaling pathway. This effect was impaired in the presence of the ERK inhibitor, PD98059 [15], indicating that EVs secreted by adipose tissue in obesity directly interfere with cancer cell signaling and functions. Both MAPK and phosphoinositide 3-kinase (PI3K)/protein kinase B (AKT) signaling pathways contribute to the proliferation and survival of breast cancer cells via vascular endothelial growth factor (VEGF), which is highly upregulated in the breast tumor microenvironment [56]. Accordingly, Rios-Colon et al. demonstrated that exosomes derived from pre-adipocytes promoted breast cancer cell tumor growth in vivo, and that the exposure of MCF-7 cells to exosomes derived from ADMSCs increased tumor progression by regulating the Wnt/β-catenin pathway. Lin et al. demonstrated that treatment of MCF-7 breast cancer cells with exosomes derived from ADMSCs promoted their proliferative rates at 48 and 72 h, but not at 24 h [57]. In contrast, exosomes derived from ADMSCs inhibited ovarian cancer cell proliferation and survival in SKOV-3 and A2780 [53,58]. Jeurissen et al. performed ex vivo experiments using real-time imaging and revealed that exosomes from breast cancer-associated adipose tissue increased the aggregation of MCF-7 cells and phosphorylation of CREB in ZR75.1 cells, suggesting a proliferative effect on breast cancer cells [55]. 

Recent studies have demonstrated that breast cancer cells deliver exosome-enriched in miRNAs to adipocytes, such as miR-144, miR-12, and miR-155, resulting in the transformation of resident adipocytes to cancer-associated adipocytes (CAA) contributing to tumor progression. In addition, miR-122 suppresses the uptake of glucose in premetastatic niche cells by decreasing the glycolytic enzyme pyruvate kinase, thus contributing to disease progression [56].

### 3.3. Inducing Angiogenesis

Angiogenesis is characterized by the formation of new blood vessels from pre-existing vascular networks via migration and proliferation of endothelial cells [59]. This process sustains tumor development through the supply of additional nutrients and oxygen, and detoxification of waste products [60]. Under physiological conditions, angiogenesis is regulated by a balance between the pro- and anti-angiogenic signaling pathways [61]. However, in tumor tissues, this balance is lost, and endothelial cells migrate and proliferate in metabolically challenging environments, such as hypoxic and nutrient-deprived tissues [62]. The adipose tissue secretome contains pro-angiogenic proteins, and the stromal vascular fractions of the adipose tissue secrete a large number of proteins involved in angiogenesis, wound healing, and tissue regeneration [63].

Pro-angiogenic factors are packaged inside exosomes [64]. Platelet-derived microvesicles, for example, are a rich source of metalloproteinases and angiogenic growth factors [65]. Gangadaran et al. demonstrated that EVs released by ADMSCs contain several angiogenic proteins, including IL-8, CCL2, and VEGF-D, which were internalized into endothelial cells. Following internalization, the endothelial cells undergo differentiation, develop a more migratory behavior, undergo a tube-like formation in vitro, and promote angiogenesis in vivo [66].

Another role of ADMSCs-derived exosomes was demonstrated by Wang et al. [67], who showed that ADMSCs treated with VEGF-C secrete exosomes enriched in miR-132. The transfer of miR-132 to lymphatic endothelial cells promotes lymphangiogenesis, a condition that occurs during tumor metastasis [68], by regulating TGF-β/Smad signaling [67].

Compared to healthy subjects, adipose tissue from patients with obesity contain higher levels of miR-31 [69], which is related to angiogenesis [70]. Kang et al. demonstrated that miR-31 is abundant in ADMSCs-derived exosomes and induces migration and tube-like formation in human umbilical vein endothelial cells (HUVECs) [71]. Based on these findings, we propose that miR-31 plays a key role in inducing angiogenesis in obesity-associated cancer.

### 3.4. Activation of Invasion and Metastasis

Invasion and metastasis are multistep processes that involve a multitude of successful biological changes in which cancer cells migrate to distant sites, triggering the formation of a premetastatic niche at secondary sites beyond the primary tumor [72]. The metastatic niche recruits immune cells and is enriched in molecular components that induce EMT in the cancer cells. Within the primary tumor, local invasion occurs first, followed by the intravasation of cancer cells into the blood and lymphatic vessels, and extravasation into the parenchyma of distant tissues, with the formation of micrometastasis, which grows into macroscopic tumors [23]. 

Khanh et al. demonstrated that in patients with type 2 diabetes mellitus, ADMSCs-derived EVs were highly associated with migration and metastasis of breast cancer cells through epidermal growth factor receptor 1 (EGFR-1)/IL-6 mediated activation of the Janus kinase (JAK)/signal transducers and activators of transcription (STAT-3) pathway [73]. In addition, these vesicles induced the upregulation of genes involved in breast cancer cell migration, such as C-X-C chemokine receptor type 4 (*CXCR4*) and *VEGF-C*, and those involved in metastasis, such as tumor growth factor β (*TGF-β*), basic fibroblast growth factor (*bFGF*), and epidermal growth factor (*EGF*). In line with this idea, we previously demonstrated that EVs derived from obese adipose tissue increase the invasive capacity of MDA-MB-231 cells through the transfer of MMP-9 [15]. Accordingly, it was previously demonstrated that exosomes from 3T3-L1 differentiated adipocytes are enriched in MMP-3 and transfer this protein to 3LL lung tumor cells, which developed increased invasive capacity through MMP-9 activation promoted by MMP-3 [74]. 

Recent studies have demonstrated a link between adipocyte EVs and tumor progression in melanoma, lung, and breast cancer [39]. Lazar et al. observed that although adipocyte-derived exosomes increased melanoma cell migration and invasion, they did not affect proliferation in vitro. Accordingly, exosomes from adipose tissue (AD-EXOs) play an important role in tumor progression in melanoma and prostate cancer, possibly through the upregulation of fatty acid oxidation (FAO) genes [75].

Lin et al. demonstrated that human ADMSC-derived exosomes increased the migration of the breast cancer MCF7 cells through the upregulation of the Wnt signaling pathway, and treatment of the cells with exosome-depleted original ADMSC-conditioned medium resulted in significantly decreased migratory capacity [57]. Wu et al. treated breast cancer MCF-7 cells with human ADMSC-derived exosomes and demonstrated that this microenvironment increased migration and invasion of the tumor cells and enhanced EMT by crosstalk between two signaling pathways: TGF-β/Smad and PI3K/AKT [76]. Furthermore, the uptake of exosomes secreted by CAA by melanoma cells allows the exchange of enzymes implicated in FAO and increases their migratory capacity [75].

In our previous study, we demonstrated that treatment of MDA-MB-231 cells with EVs derived from adipose tissues of patients with obesity led to increased migration and invasiveness via PI3K/AKT dependent signaling. The migratory and invasive effects were impaired in the presence of the PI3K/AKT inhibitor, LY294002 [15]. 

Interestingly, supporting evidence of the bidirectional crosstalk between adipose tissue and breast cancer cells has also been demonstrated previously. Breast cancer-derived exosomes were shown to promote a myofibroblastic phenotype in adipose tissue-derived mesenchymal stem cells, resulting in increased expression of the tumor-promoting factors, *TGF-β*, *VEGF*, stromal cell-derived factor 1 (*SDF-1*), and *CCL5* [14]. 

### 3.5. Dysregulation of Cellular Energetics

Alterations in the metabolism in tumor cells is a common trait that favors the growth and proliferation of cancer cells. Competition in the tumor microenvironment induces these cells to acquire a phenotype that requires high uptake of glucose by upregulation of the glucose transporter (GLUT) receptors [77]. However, the increased concentration of glucose in these cells is mostly utilized for glycolysis, even in the presence of oxygen, through a mechanism called aerobic glycolysis (Warburg effect), which promotes the production of lactate, decreases pyruvate concentration, and negatively affects oxidative phosphorylation in the citric acid cycle [78]. This dysregulation of the energetic complexes in the cells uses glycolysis to bypass some intermediate molecules to synthesize biomolecules via the pentose phosphate pathway, fostering the metabolism of tumor cells. Subsequently, the tumor microenvironment often becomes deficient in oxygen, resulting in the activation of transcription factors, such as HIF1α, thereby promoting the activity of the glycolytic enzymes [23].

Recent studies have demonstrated the role of adipose tissue EVs in communication with melanoma, breast, and ovarian cancer cells, modulating their metabolism and enhancing some malignant characteristics [8]. For example, enzymes involved in fatty acid metabolism, such as the trifunctional enzymes and hydroxyacyl-coenzyme A dehydrogenase, were found within adipocyte-derived exosomes. They act on the fatty acid oxidative cycle in neoplastic cells, improving lipid metabolism and respiratory chain activity, and tumor cell migration in melanoma [75]. Adipocytes maintain crosstalk with cancer cells, fueling the tumor microenvironment with free fatty acids, leptin, ketone bodies, and other macromolecules that alter the metabolism in neoplastic cells. These free fatty acids enter tumor cells via lipid transporters such as fatty acid-binding protein (FABP4) and fatty acid transport protein (FATP1) [39]. Such alterations in lipid availability favor β-oxidation and leptin activation of PI3K/MAPK and JAK/STAT pathways, promoting cell proliferation and survival [9]. Furthermore, the Warburg effect can also be induced by glycolytic enzymes transported from bone marrow adipocytes to cancer cells. Following uptake by the cells, the cells undergo oxidative stress and secrete reactive oxygen species (ROS) into the tumor microenvironment, promoting the “reverse Warburg effect” to trigger aerobic glycolysis and production of high energy metabolites [39]. Clement et al. demonstrated that AD-EVs provide fatty acids to the melanoma microenvironment that are stored in lipid droplets, and are used to maintain fatty acid oxidation (FAO) in mitochondria. In addition, EVs containing proteins related to mitochondrial dynamics, including mitochondrial fission protein 1 (FIS-1), and mitochondrial dynamin-like GTPase 1 (OPA-1) were found to induce, together with FAO, mitochondrial redistribution to the edge of melanoma cells, favoring migration and consequently melanoma aggressiveness [45]. Accordingly, it has been demonstrated that cellular growth and metastasis are mediated by metabolic remodeling in adipocytes surrounding the ovarian [41] and breast cancer cells [79]. Moreover, carbohydrates and amino acids are also present in exosome traffic between neoplastic cells and tumor-associated adipose tissue, sustaining the energy and substrates for the tumor microenvironment by increasing metabolic pathways [56]. MiRNAs found in exosomes, including miR-105, miR-122, miR-126, and miR-155, play a key role in reprograming the energy metabolism in breast cancer cells and CAA [56]. Wu et al. demonstrated that adipocytes undergo a marked reduction in their lipid content following coculture with breast cancer cells [76], demonstrating that tumor-surrounding adipocytes are lipolytic. When co-cultured with adipocytes, MDA-MB-231 and MCF-7 cells became enriched in lipid droplets, and showed increased uptake of glucose and FAO enzymes. Furthermore, the close relationship between adipocytes and tumor cells induced the uptake of tumor exosomes enriched in miR-144 and miR-126 by adipocytes, which mediated beige/brown differentiation of adipocytes and metabolic remodeling, respectively. When both miRNAs were blocked, adipocyte-induced tumor growth was reduced [80], reinforcing that cell-to-cell communication between adipocytes and tumor cells plays a pivotal role in breast cancer progression. Such alterations are increased in obese individuals due to a larger adipose tissue reservoir and exosomal traffic, resulting in poorer outcomes in cancer.

### 3.6. Enabling of Replication Immortality

Telomeres protect the ends of chromosomes and play a pivotal role in cell proliferation. Following shortening of telomeres, cells become senescent and progressively lose their proliferative capacity. The activation of human telomerase reverse transcriptase (hTERT) induces resistance to apoptosis and senescence [23], and is one of the key molecular events in tumor initiation and progression in many tissues such as the liver [81,82]. Gutkin et al. demonstrated that exosomes from Jurkat leukemia cancer cells were enriched in *hTERT* mRNA and, once they are shuttled via exosomes to telomerase negative fibroblasts, these cells showed increased hTERT expression and activity, and increased cell proliferation and life span [83]. 

Stefanou et al. demonstrated that following leptin stimulation, HepG2 hepatocellular carcinoma cells exhibited hTERT upregulation via the JAK/STAT-3 pathway and Myc/Max/Mad network, which demonstrates that leptin plays a role in HCC cell proliferation and progression [84]. Accordingly, leptin has been linked to the induction of telomerase activity via STAT-3 in MCF-7 breast cancer cells [85].

Sun et al. demonstrated that miR-27a is highly upregulated in cancer, plasma, and adipose tissue samples in obese patients with liver cancer compared to non-obese patients with cancer. Furthermore, they demonstrated that co-culturing 3TL-L1 adipocytes overexpressing miR-27a with HepG2 cancer cells downregulated forkhead box protein O1 (FOXO1) and promoted G1/S cell cycle transition in HepG2 cells by decreasing p21 and p27 and increasing cyclin D1 levels [86]. Given that miR-27a is released by adipose tissue within exosomes [87], it is reasonable that miR-27a enriched AD-Exos could stimulate liver cancer development.

Even though there is no clear evidence demonstrating the direct effect of adipose tissue-derived EVs on enhancing telomerase activity, EVs released by obese adipose tissue, enriched in leptin, underlie the activation of hTERT, with possible interconnection between adipose tissue and shortening of telomeres.

### 3.7. Evading Growth Suppressors, Gene Instability, and Mutation

Normal tissue architecture and function are maintained by the elimination of damaged cells, and cellular quiescence must be overcome to establish a neoplastic lesion. Tumor suppressors limit cell cycle transitions by blocking the progression from G1 to S phase of the cell cycle [88,89]. Tumor suppressors, including retinoblastoma protein (Rb), p53, Myc, and Warts/Hippo, operate as master regulators of circuits governing cell proliferation or apoptosis [23,90]. However, certain conditions mitigate the activity of tumor suppressors. For example, in MCF-7 breast cancer cells, miR-155 targets TP531NP1, conferring resistance to cell death [51]. Given that miR-155 is the main miRNA found in exosomes released by adipose tissue macrophages in obesity [49], we speculate that such exosomes play a role in the evasion of growth suppression through inhibition of p53. Wang et al. demonstrated that treatment of MCF-7 breast cancer cells with exosomes from ADMSCs led to the activation of several signals including that of yes-associated protein (YAP) and taffazin (TAZ), two key downstream effectors of Hippo signaling, one of the major pathways controlling tumorigenesis [47]. Treatment of MCF-7 cells with the conditioned media derived from obese adipose tissue led to decreased p27 and hypophosphorylated Rb, likely due to the action of leptin [91]. However, further studies focusing on the role of leptin-enriched exosomes derived from adipose tissue are needed to better understand these processes, given that these obese AT-EVs are enriched in leptin [15,92]. 

Alterations in the genome of neoplastic cells drive and sustain the acquisition of cancer hallmarks, enabling their outgrowth in the tissue microenvironment and subsequent clonal expansion. Cancer stem cells are characterized by mutability, and the rates of mutation are often very high to escape systems that detect and resolve defects in the DNA. Reinforcing the role of exosomes from adipose tissue in this process, it was previously demonstrated that miR-155, the main miRNA found in exosomes from adipose tissue macrophages, inhibits p53 in MCF-7 cells [51].

Wu et al. reported that, following uptake of exosomes derived from the co-cultivation of MDA-MB-231 cells and adipocytes, the adipocytes showed increased levels of miR-144 and miR-126, suggesting their differentiation toward a beige/brown phenotype associated with pro-tumorigenic process, showing that specific miRNAs within adipose tissue-derived exosomes are associated with the pro-tumorigenic process [80]. Another study demonstrated that exosomes secreted from 3T3-L1 pre-adipocytes contain SRY-box transcription factor 9 (SOX-9), which promotes tumorigenesis in MCF-10 epithelial cells in vitro [93]. These results highlight the role of adipose tissue-derived exosomes in signal transduction within the tumor microenvironment. Furthermore, exosomes derived from obese adipose tissue may present the tumor suppressors miR-148b and miR-4269 downregulated, while oncomiR and miR-23b may be upregulated [94], suggesting that exosomes from obese adipose tissue increase the risk of neoplastic transformation.

### 3.8. Avoiding Immune Destruction and Inflammation

Traditionally, immune cells, such as neutrophils, macrophages, and T cells, are considered to be protective mechanisms of the body, that help in eliminating microbial infections and potential cancer cells. However, instead of fighting infection and healing, inflammatory cells can have their functions modified, thereby supporting and enabling the acquisition of hallmark capabilities, favoring tumor growth [95,96,97]. Inflammation may supply the tumor microenvironment with bioactive molecules such as chemicals and ROS, which are highly mutagenic for the surrounding cancer cells, enhancing their genetic evolution toward increased malignancy. Therefore, inflammation is considered an enabling characteristic for its contribution to the acquisition of core hallmark capabilities [23].

In obesity, adipose tissue expansion caused by hyperplasia and hypertrophy of adipocytes leads to the release of proinflammatory adipokines [15], sharing similarities with the tumoral microenvironment where inflammation underlies tumor growth [11].

Adipocytes present in the tumor microenvironment release EVs that suggests that obesity is a risk factor for the progression of cancer and poor prognosis. Tumors that are usually located in the foci of adipose tissues such as breast, ovarian, and melanomas prepare what is called a proinflammatory niche, which is the main event that gives the tumor the ability to form colonies to reach new tissues and metastasize [8,98]. Sun et al. demonstrated that adipose tissue is directly involved in chronic inflammation, leading to an increase in adipocyte hyperplasia and cytokine-related signaling pathways (IL-6, IL-8, and C-C chemokine receptor type 5 (CCR5) in macrophages) within the breast cancer microenvironment. In addition, they showed that macrophage infiltration in adipose tissue precedes the development of breast tumors [99]. Similarly, Nieman et al. demonstrated that adipocytes from the omental tissue promote migration and invasion of ovarian tumor cells, which is mediated by adipokines such as IL-8 [41]. The observation that macrophages may be part of the formation of tumors is not novel. There are reports in the literature, from the late 1970s, that these cells called tumor-associated macrophages (TAMs), contribute to tumor growth [100].

During the early stages of cancer, TAMs generally have immunostimulatory functions [101]. In the later stages of tumor progression, the tumor microenvironment becomes rich in growth factors and mediators such as IL-4, IL-10, and TGF-β, which mediate macrophage polarization and acquire the M2-like phenotype [102]. M2-like macrophages lack activity and phagocytic capacity and produce and secrete growth factors (fibroblast growth factor (FGF), macrophage-colony stimulating factor (M-CSF), platelet-derived growth factor (PDGF), TGF-β, and VEGF) in the microenvironment, in addition to presenting a more immunosuppressive profile [103,104,105,106].

Zhao et al. showed that murine exosomes derived from AT-MSCs are internalized by macrophages, leading to an increase in the mRNA levels of Arginase-1 and IL-10. These vesicles also transfer phosphorylated STAT-3, which together with an increase in arginase-1 content, induce macrophage polarization toward an M2 phenotype [107]. It is important to note that within the tumor microenvironment, TAMs have characteristics similar to that of M2 macrophages [103,104,105,106]. Therefore, we propose that exosomes derived from AT-MSCs may improve tumor growth through macrophage M2-like polarization.

The overexpression of programmed cell death ligand 1 (PD-L1) triggers inhibitory signaling in cytotoxic T cells, suppressing its antitumor effects [108]. In a mouse model of obesity, Li et al. demonstrated that tumoral expression of PD-L1 is increased together with a decrease in CD8^+^ and exhaustion of tumor-infiltrating lymphocytes in hepatocarcinoma and melanoma. Interestingly, they demonstrated that TNF-α and IL-6 secreted by adipocytes sustained this effect, once both the cytokines neutralized the expression of PD-L1 [109].

Blazquez et al. showed that human exosomes derived from AT-MSCs lead to a decrease in interferon-gamma secretion and a decrease in MHC class II, leading to the inactivation of T cells (CD4^+^ and CD8^+^) [110].

Chronic inflammation together with adipose enlargement induced by obesity leads to an “exhausted” T cell phenotype, which is associated with PD-1 upregulation [111]. Accordingly, it has been demonstrated that T cells present in the melanoma and breast carcinoma microenvironment have features of exhaustion, together with a decrease in tumor-infiltrating CD8^+^ T cells driven by the upregulation of carnitine palmitoyltransferase I (*Cpt1a*) gene expression, a master regulator of FAO. Interestingly, when leptin signaling was blocked, both increased PD-1 expression and exhaustion of CD8^+^ T cells were restored [111]. Considering that EVs derived from obese adipose tissue contain IL-6 [92,112], TNF-α [113], and leptin [15,92], these EVs likely play a role in CD8^+^ T cells, thus contributing to the evasion of immune destruction.

Wu et al. demonstrated that CAA release increased adipokines, including IL-6, TNF-α, CCL-2, and CCL-5, promoting invasion and metastasis of breast cancer [56]. Such adipokines have been detected in EVs, thus suggesting a role for adipose tissue-derived EVs in the communication between adipocytes and breast cancer cells within the tumor microenvironment.

## 4. Dual Roles of AT-EVs in Tumor Cells: miRNAs

MiRs are involved in physiological and pathological processes [114]. They are transported inside EVs that provide protection against degradation (different from when they are released directly into the body fluids or the circulation), and mediate intercellular communication with cells in distant organs, in a systemic manner [115,116].

However, how exosomes containing miRNAs affect cancer cells is not well established. Singh et al. showed that exosomes derived from MDA-MB-231 breast cells are internalized by HMLE breast epithelial cells, inducing invasive ability. These results suggest that non-invasive cells can internalize miR-enriched exosomes derived from tumor cells that then change their characteristics [117]. In another study, Li et al. observed that exosomes containing miR-1246 are shuttled from MDA-MB-231 cells to epithelial breast cells. Furthermore, these exosomes induce cell proliferation and migration in non-tumoral cells. These effects are associated with miR-1246, which targets CCNG2 expression [118].

Thomou et al. demonstrated that adipose tissue is the major source of circulating EVs containing miRNAs (miR-EVs). Lower levels of miR-exosomes have previously been reported in patients with lipodystrophy [119], reaffirming the importance of miR-EVs derived from adipose tissue.

MiR plays an important role in several types of cancers [120,121,122], and studies have shown that miR-EVs derived from hypoxic HepG2 cancer cells, which are enriched in miR-23, modulate the microenvironment to facilitate tumor progression by increasing angiogenesis, a hallmark of cancer. However, not only are tumor cells capable of releasing miR-EVs, but miR-EVs released by adipose tissue have been gaining prominence in cancer progression [123].

MiR-23 is an oncomir that is capable of promoting cancer malignancy [124,125]. Liu et al. demonstrated in vivo that patients with a high body fat ratio exhibited miR-23a/b upregulation in serum exosomes and tumor tissues. Additionally, they further demonstrated, in vitro, that such exosomes when delivered to hepatocellular carcinoma (HCC) cells, induce their proliferation, migration, and chemoresistance through the von-Hippel-Lindau (VHL)/HIF-1α axis [126].

Circular RNAs (circRNAs) are a novel type of non-coding RNA [127] which are capable of absorbing miRNAs by stable complementary binding [128]. Zhang et al. reported that exosomes derived from adipocytes presented increased levels of circRNAs, which were transferred to HCC cells in both an in vitro and an in vivo mouse model, inducing proliferation and decreased DNA damage through the suppression of miR-34a and activation of cyclin A2 [129].

Bonmarrow adipose tissue (BMAT) is recognized as a key contributor to multiple myeloma (MM) oncogenesis and progression [130]. It is believed that this interaction is mediated by miR-EVs, since, in obesity, there is an increase in miR-EVs secreted by BMAT cells, which are involved in the poor prognosis of MM, together with a decrease in tumor suppressor miRNAs [131].

Wu et al. reported that when breast cancer cells were co-cultured with mature adipocytes, they underwent upregulation of miR-144 and miR-126 expression, which were further released within EVs, triggering beige/brown differentiation in white adipocytes together with the release of free fatty acids, lactate, and pyruvate, which are required for tumor proliferation and metastasis [80]. Several other studies support a close association between miRNAs derived from adipose tissue and liver and prostate cancer progression as well as in cancer-associated cachexia [86,132,133]. Although these studies did not directly state that these miRNAs were released from adipose tissue into EVs, we cannot disregard their roles in these studies.

In contrast, miR-EVs can exert a dual role, and also exhibit antitumor action. Takahara demonstrated that miR-134, which was previously described as a tumor suppressor, is present in ATMSC-derived EVs and suppresses the growth of prostate cancer cells by inducing apoptosis via increased activity of B-cell lymphoma-extra large (Bcl-xL) [134]. Eexosomes derived from human adipose-derived mesenchymal stem cells are enriched in miRNAs with anti-cancer activities. These exosomes impair ovarian cancer cell proliferation by inducing apoptosis and blocking the cell cycle through the upregulation of different pro-apoptotic molecules [48]. Furthermore, it has been demonstrated that intra-tumor injection of ATMSC-Exos enriched in miR-122 enhanced the chemosensitivity of HCC cells to chemotherapeutic agents in vivo [135].

The global repression of miRNA maturation triggers cellular transformation and tumorigenesis [136]. Kim et al. demonstrated that in subcutaneous and visceral adipose tissue depots of obese patients, miRNAs (miR-133a, miR-139-5-p, miR-26a, and miR-451) were downregulated, while miR-155 was upregulated [137]. Studies have shown that these miRNAs, which are downregulated in adipose tissue depots from obese patients, have a well-established tumor suppressive activity [138,139,140,141]; whereas miR-155, which was upregulated in the same depots, serves as an oncomir [142].

Considering that most circulating miRNAs within exosomes are released from adipose tissue depots, we propose a critical role for healthy adipose tissue in maintaining homeostasis and an anti-cancer role through miRNAs released within EVs, which is disturbed in obesity. In obese adipose tissues, a large number of miRNAs with tumor suppressor activities are downregulated and miRNAs with pro-tumor roles are upregulated, raising the possibility that the inflammatory changes occurring in the obese adipose tissue favor the establishment of tumors and portend poor prognosis.

Zhang et al. demonstrated that ATMSC-EVs enriched in miR-101 suppressed lung metastasis of osteosarcoma in a nude mouse model, demonstrating the potential effectiveness of AD-EVs in cancer malignances [143]. Regarding obesity, Ortega et al. demonstrated that miR-101 is downregulated in pre-adipocytes and mature adipocytes [144].

Therefore, these data suggest that miR-EVs from adipose tissue may be therapeutic targets for cancer. Therefore, it is plausible to propose that the use of EVs derived from healthy adipose tissue may create a growth-inhibitory environment and provide a better prognosis for cancer.

## 5. Perspectives on Immune Cells

### 5.1. Tumor-Associated Neutrophil Polarization to N1 Phenotype

Neutrophils have phenotypic and functional heterogeneity, which changes according to the microenvironment [145,146,147], and the number of studies investigating the role of neutrophils in tumor progression is increasing [148,149,150]. The first studies in mice reported that depletion of neutrophils or blocking their migration decreased tumor growth [151,152]. In humans, several studies have shown that in various types of cancers, the density and localization of neutrophils in the tumor microenvironment are associated with a poorer survival rate [153,154,155,156]. However, the presence of neutrophils is associated with a good prognosis in patients with advanced gastric carcinoma [157].

Similar to macrophages, tumor-associated neutrophils (TANs) may exhibit two different phenotypes: cytotoxic (N1) and immunosuppressive (N2) [151,158,159]. N1 produces high amounts of TNF-α and ROS, and has an increased phagocytic capacity, resulting in a decrease in tumor growth [151]. In contrast, N2 presents a pro-tumor profile that contributes to tumor growth, angiogenesis, and metastasis [160,161]. Furthermore, the contribution of TANs to tumor development is dependent on the tumor stage [162].

Our group has shown that human adipose tissue releases microparticles derived from pre-adipocytes, adipocytes, neutrophils, and other leukocytes [10], which affects breast cancer cells [15], and could affect the tumor microenvironment.

Mahmoudi et al. showed that ADMSCs produce exosomes that are internalized by human neutrophils. These EVs decrease neutrophil apoptosis and increase phagocytic capacity [163]. In agreement, these data suggest that ADMSC-derived exosomes shift neutrophil polarization toward an N1-like profile, which may be a potentially interesting strategy for cancer treatment.

### 5.2. Lymphocytes

As mentioned previously, PD-L1, present in many cancer cells, binds to the PD-1 receptor on T cells, leading to suppression of antigen-derived activation and checkpoint response in these cells [108]. Patients with melanoma and head and neck cancers have exosome-enriched PD-L1 in plasma samples [164,165]. In addition to PD-L1-enriched exosomes being considered as a biomarker for cancer, we could consider the use of anti-PD-1 therapy [164]. In this context, Zhou et al. observed that human exosomes derived from AT-MSCs are rich in miR-424-5p when compared to exosomes derived from MSCs. The authors treated MDA-MB-231 breast cancer cells with exosomes derived from AT-MSCs enriched with miR-424-5p and observed downregulation of PD-L1 expression. Tumor cells that received these exosomes presented high levels of apoptosis when co-cultured with T cells. Finally, when miR-424-5p was delivered via exosomes intratumorally in mice, breast cancer grew slowly, and tumor growth was strongly suppressed [166]. Taking these data into consideration, treatment with exosomes derived from AT-MSCs enriched with miR-424-5p represents an interesting strategy to induce cytotoxic T cells in tumor cells.

## 6. Conclusions and Future Perspectives

In this review, we provide evidence showing that the EVs secreted by adipose tissue may carry pro-tumor molecules, which can modulate the cancer cells behavior and functions described as the hallmarks of cancer (Table 1). Recent findings have demonstrated a close relationship between EVs released by adipose tissue and the establishment and progression of several types of cancer. Such communication may occur both practically and systemically. Various cell types within the adipose tissue can release these EVs, such as adipocytes, pre-adipocytes, macrophages, and mesenchymal stem cells. Furthermore, cell-to-cell communication mediated by EVs occurs bidirectionally. For example, EVs released by adipose tissue can fuel tumor cell metabolism and induce tumorigenesis, tumor progression, and metastasis. In addition, EVs released by tumor cells can also modify the phenotype and metabolic profile of adipocytes, which undergo a pro-tumoral behavior that sustains tumor growth via EVs.

During obesity, the cargo released within these vesicles is altered with increased amounts. Conversely, EVs from healthy adipose tissue are enriched in tumor suppressor molecules, such as miRNAs, promoting the apoptosis of tumor cells. Thus, it is conceivable to postulate that: (i) EVs from healthy adipose tissue may offer benefits against tumor development, and (ii) EVs from obese adipose tissue play a pivotal role in cancer establishment and progression. Understanding the regulatory pathways governing the biogenesis, secretion, and cargo selection of EVs derived from adipose tissue may be a useful strategy to thwart tumorigenesis and metastasis in obese patients.

Therefore, blocking the enlargement and inflammation within adipose tissue as well as understanding the pathways involved in extracellular vesicle release and cargo selection are prominent strategies in the battle against cancer.

## Figures and Tables

**Table 1:** Extracellular vesicles released by the adipose tissues and the modulated hallmarks.

Resisting Cell Death			
Type of Vesicle	Content	Effect	References
ADMSC-Exos	N.D.	Protect breast cancer from apoptosis	[47]
ATM-Exos	miR-155	Resistance to cell death in breast cancer cells via caspase-3, Bcl-2, APAR-1, FADD, and RIP1	[50]
AT-Exos	Decreased levels of miR-148b	Decrease apoptosis in breast cancer cells	[21]
**Sustaining Proliferative Signaling**			
**Type of vesicle**	**Content**	**Effect**	**References**
AT-Exos	N.D.	Tumor progression by reprogramming surrounding cells/ Increases proliferation and invasion/ Increase angiogenesis	[54]
BCC-EVs	miR-144 miR-126 miR-155	Increased lipolysis in adipocytes tissue toward CAA phenotype	[56]
AD-Exos	N.D.	Increased MMP-3 in lung cancer cells	[54]
CAA-Exos	miR-21	Suppressed ovarian cancer cells apoptosis and increased chemoresistance by targeting APAF-1	[129]
AT-Exos (obese AT)	circ-DB RNAs	Growth of hepatocarcinoma by targeting USP-7 deubiquitination	[129]
ADMSC-Exos	N.D.	Increased proliferation of osteosarcoma cells via induction of COLGALT2	[39]
AT-EVs (obese AT)	N.D.	Increased CREB phosphorylation in ZR75.1 breast cancer cells	[55]
AD-Exos	miR-23	Increased cell growth of hepatocarcinoma cells	[124]
AT-EVs (obese AT)	N.D.	Increased proliferation of MCF-7 breast cancer cells via ERK phosphorylation	[15]
**Inducing Angiogenesis**			
**Type of Vesicle**	**Content**	**Effect**	**References**
ADMSC-EVs	IL-8 CCL2 VEGF-D	Increased migration and tube-like formation in endothelial cells	[66]
ADMSC-Exos treated with VEGF-C	miR-132	Lymphangiogenesis via TGF-β/Smad signaling	[67]
ADMSC-Exos	miR-31	Increased migration and tube-like formation in (HUVEC) endothelial cells	[70]
**Activating Invasion and Metastasis**			
**Type of Vesicle**	**Content**	**Effect**	**References**
ADMSC-EVs from diabetic subjects	EGFR-1/IL-6	Increased migration and metastasis of breast cancer cells via EGFR-1/IL-6 activating JAK/STAT-3 pathway	[73]
AT-EVs (obese AT)	MMP-9	Increased invasive capacity of MDA-MB-231 breast cancer cells	[15]
AD-Exos (from differentiated 3T3-L1)	MMP-3	Increased invasive capacity of 3LL lung tumor cells	[74]
AD-EVs	N.D.	Tumor progression in melanoma, lung, and breast cancer	[39]
AD-Exos	N.D.	Increase in melanoma cell migration and invasion; tumor progression in melanoma and prostate cancer by upregulating genes involved in fatty acid oxidation	[75]
ADMSC-Exos	N.D.	Increased migration of MCF-7 cells by through the upregulation of Wnt- signaling pathway	[57]
ADMSC-Exos	N.D.	Increased migration, invasion, and epithelial-mesenchymal transition of MCF-7 cells by TGF-β/Smad and PI3K/AKT signaling pathways crosstalk	[76]
CAA-Exos	N.D.	Exchange of enzymes implicated in fatty acid oxidation triggering melanoma cells migration.	[45]
AT-EVs (obese AT)	N.D.	Increased migratory capacity in MDA-MB-231 breast cancer cells via PI3K/AKT signaling pathway	[15]
AD-Exos	miR-23	Increased migration in HCC cells through VHL/HIF-1α axis	[126]
**Dysregulation of Cellular Energetics**			
**Type of Vesicle**	**Content**	**Effect**	**References**
AD-Exos	Hydroxyacyl-coenzyme A dehydrogenase (HCDH)	Improving lipid metabolism, respiratory chain activity, and tumor migration in melanoma cells	[75]
AD-Exos	Mitochondrial fission protein 1 (FIS-1) Mitochondrial dynamin like GTPase (OPA-1)	Induce mitochondrial redistribution to the edge of melanoma cells favoring migration	[45]
AD-Exos	Trifunctional enzyme (ECHA)	Increased fatty acid oxidation in melanoma cells	[45]
**Evading Growth Suppressors**			
**Type of Vesicle**	**Content**	**Effect**	**References**
ADMSC-Exos	N.D.	Breast cancer cell growth via activation of YAP and TAZ downstream Hippo signaling pathway	[47]
**Gene Instability and Mutation**			
**Type of Vesicle**	**Content**	**Effect**	**References**
ATM-Exos	miR-155	Inhibited p53 in MCF-7 cells	[51]
AD-Exos	SOX-9	Induced tumorigenesis in MFC-10 epithelial cells	[93]
**Avoiding Immune Destruction and Inflammation**			
**Type of Vesicle**	**Content**	**Effect**	**References**
ADMSC-Exos	N.D.	Inactivation of T cells	[110]

ADMSC-Exos: exosomes derived from adipose tissue mesenchymal stem cells; ADMSC-EVs: extracellular vesicles derived from adipose tissue mesenchymal stem cells; ATM-Exos: exosomes derived from adipose tissue macrophages; AD-Exos: exosomes derived from adipocytes; AD-EVs: extracellular vesicles derived from adipocytes; AT-EVs: extracellular vesicles derived from adipose tissue; CAA-Exos: exosomes derived from cancer-associated adipocytes; AT-Exos: exosomes derived from adipose tissue; BCC-EVs: extracellular vesicles derived from breast cancer cells.

## Data Availability

Not applicable.
